# Exposure of Asian Elephants and Other Exotic Ungulates to Schmallenberg Virus

**DOI:** 10.1371/journal.pone.0135532

**Published:** 2015-08-14

**Authors:** Fieke M. Molenaar, S. Anna La Rocca, Meenakshi Khatri, Javier Lopez, Falko Steinbach, Akbar Dastjerdi

**Affiliations:** 1 Zoological Society of London, ZSL Whipsnade Zoo, Bedfordshire, United Kingdom; 2 Virology department, Animal and Plant Health Agency (APHA)-Weybridge, Woodham Lane, Addlestone, Surrey, United Kingdom; 3 Chester Zoo, Upton-by-Chester, Cheshire, United Kingdom; Institut National de la Santé et de la Recherche Médicale (INSERM), FRANCE

## Abstract

Schmallenberg virus (SBV) is an emerging Orthobunyavirus, first described in 2011 in cattle in Germany and subsequently spread throughout Europe, affecting mainly ruminant livestock through the induction of foetal malformations. To gain a better understanding of the spectrum of susceptible species and to assess the value of current SBV serological assays, screening of serum samples from exotic artiodactyls and perissodactyls collected at the Living Collections from the Zoological Society of London (Whipsnade and London Zoos) and Chester Zoo was carried out. There was compelling evidence of SBV infection in both zoological collections. The competitive ELISA has proved to be applicable for the detection of SBV in exotic *Bovidae*, *Cervidae*, *Suidae*, *Giraffidae* and most notably in endangered Asian elephants (*Elephas maximus*), but unreliable for the screening of *Camelidae*, for which the plaque reduction neutralisation test was considered the assay of choice.

## Introduction

Schmallenberg virus (SBV) is an emerging Orthobunyavirus affecting mainly ruminant livestock through the induction of foetal malformations. It was first described in the late summer of 2011 in cattle in Germany that had a sudden drop in milk yield [1], in the autumn of 2011 in congenitally malformed sheep in the Netherlands [2], but infection was also demonstrated in wild cervids through retrospective serology in Fall 2011 in Belgium [3]. The virus has subsequently spread throughout Europe [4], and was first detected in the UK in early 2012 [5]. SBV is an Orthobunyavirus [1] transmitted by biting arthropods, and *Culicoides* midges have been identified as key vectors for its spread [6–11]. In line with similar vector-borne diseases, such as Bluetongue, evidence suggests windborne incursion of the virus into the UK via infected midges, and not via imported livestock [12]. SBV antibodies have been detected in various wild ungulates throughout Europe [13–16], including red deer (*Cervus elaphus*) [13–16], fallow deer (*Damma damma*) [15], chamois (*Rupicapra rupicapra*) [13], moose (elk, *Alces alces*) and European bison (*Bison bonasus*) [16].

Despite the detection of SBV-induced antibodies in wild and captive ruminant species mentioned above, there is an under-appreciation of the spectrum of susceptible species, due to the very short viraemia and a lack of clinical symptoms in the acute phase of the infection. This study was therefore aimed to address this by examining archived sera from exotic ungulates collected from the Zoological Society of London (ZSL) Whipsnade and London Zoos and Chester Zoo. In addition, the value of current SBV serological assays, a competitive ELISA (cELISA) and a plaque reduction neutralisation test (PRNT), for screening serum samples from artiodactyls and perissodactyls was also assessed.

## Materials and Methods

### Ethical statement

All samples used for this study were surplus archived serum samples, initially collected for clinical purposes under the Veterinary Surgeons Act 1966, United Kingdom.

### Archived serum sample selection

Frozen serum samples stored at -20°C from *Artiodactyla*, *Perissodactyla* and *Proboscidea* collected at ZSL Whipsnade Zoo (Collection A) between January 1, 2011 and February 1, 2014 (211 samples from 136 individuals of 39 species) and at Chester Zoo (Collection B) between August 1, 2012 and December 1, 2013 (28 samples from 28 individuals of 16 exotic ungulate species) were used for this study. Two further samples from two camelid species from ZSL London Zoo (Collection C) were also included. In total, 241 samples from 165 individuals of 48 different exotic ungulate species were tested using the cELISA and 35 samples from 34 individuals of 20 different species were further verified using PRNT (Table 1).

**Table 1:** List of serum samples used in this study, detailing number of individual species and samples tested with cELISA and PRNT. The two tests were carried out on archived sera collected from exotic wildlife species in the *Bovidae*, *Cervidae*, *Suidae*, *Giraffidae*, *Camelidae* and *Elephantidae* families. The sera were from one to several individual animals of each species, All but one were tested with cELISA, and a random selection of cELISA positives and negatives were further investigated using PRNT.

Species—Latin name	No. of samples/Individuals	cELISA/PRNT
Impala—*Aepyceros melampus*	2/2	2/0
Moose—*Alces alces*	5/5	5/0
Blackbuck—*Antilope cervicapra*	1/1	1/1
American bison—*Bison bison*	1/1	1/0
European bison—*Bison bonasus*	9/7	9/0
Gaur—*Bos gaurus*	2/2	2/1
Yak—*Bos grunniens*	19/11	16/5
Banteng—*Bos javanicus*	1/1	1/0
Goats—*Capra aegagrus hircus*	4/4	4/0
Red forest duiker—*Cephalophus natalensis*	1/1	1/0
Roan antelope—*Hippotragus equinus*	1/1	1/0
Waterbuck—*Kobus ellipsiprymnus*	2/2	2/0
Nile lechwe—*Kobus megaceros*	4/2	3/1
Scimitar-horned oryx—*Oryx dammah*	19/16	19/2
Gemsbok—*Oryx gazella gazella*	10/6	10/0
Arabian oryx—*Oryx leucoryx*	4/4	4/0
Congo buffalo—*Syncerus caffer nanus*	2/2	2/0
Lesser kudu—*Tragelaphus imberbis*	3/3	3/0
Bongo—*Tragelaphus eurycerus*	5/4	5/0
Sitatunga—*Tragelaphus spekii*	15/11	15/0
Greater kudu—*Tragelaphus strepsiceros*	5/3	4/3
Bactrian camel—*Camelus bactrianus*	6/6	6/1
Llama—*Lama glama*	4/3	4/2
Alpaca—*Vicugna pacos*	2/2	2/1
Hog deer *Axis—Hyelaphus porcinus*	3/2	2/2
Axis deer—*Axis axis*	1/1	1/0
Fallow deer—*Dama dama*	1/1	0/1
Pere David's deer—*Elaphurus davidianus*	4/4	4/0
Chinese water deer—*Hydropotes inermis*	1/1	1/0
Brow antlered deer—*Panolia eldii*	1/1	1/0
Reindeer—*Rangifer tarandus*	1/1	1/1
Swamp deer—*Rucervus duvaucelii*	4/4	4/0
Philippine spotted deer—*Rusa alfredi*	2/1	2/0
Reticulated giraffe—*Giraffa c*. *reticulata*	5/3	5/2
Rothschild giraffe—*Giraffa c*. *rothschildi*	3/3	3/0
Pygmy hippo—*Hexaprotodon liberiensis*	3/3	3/0
Babirusa—Babyrousa celebensis	2/2	2/2
Red river hog—*Potamochoerus porcus*	2/2	2/1
Domestic donkey—*Equus africanus asinus*	2/2	2/0
Poitou donkey—*Equus asinus Poitou*	2/2	2/0
Shetland pony—*Equus ferus caballus*	2/2	2/0
Przewalski's horse—*Equus ferus przewalskii*	5/2	5/0
Grevy's zebra—*Equus grevyi*	14/7	14/1
Onager—*Equus hemionus*	10/7	10/4
White rhinoceros—*Ceratotherium simum*	4/4	4/1
Indian rhinoceros—*Rhinoceros unicornis*	2/2	2/0
South American tapir—*Tapirus terrestris*	1/1	1/0
Asian elephant—*Elephas maximus*	37/10	37/3

### ELISA

The cELISA ‘ID Screen Schmallenberg virus Competition Multi-species’ (ID.vet) was used for the initial serological screening, according to manufacturer’s instructions. The ratio of the OD of the sample (S) to the OD of the negative control (N) was subsequently calculated to obtain the S/N %. The serum was considered positive for SBV antibodies when the S/N % was smaller than or equal to 40 and negative when the S/N % was more than 50. Test results between these measures were considered inconclusive.

### PRNT

To validate the results obtained from the cELISA and to quantify the levels of SBV neutralising antibodies, samples with an S/N % of 40–60 and a random selection of positives and negatives from a variety of species were tested using PRNT [17]. For this test, a 2-fold dilution series of the serum was mixed with a standard quantity of SBV and incubated on a monolayer of cells. Non-neutralised virus leads to cytopathic effect (cpe) in the infected cells. The resultant plaques were visualised following fixation and staining of the monolayer. The number of plaques is inversely proportional to the level of antibodies present in the tested sera.

Briefly, the assay was carried out using Vero cells (kidney epithelial cells from *Chlorocebus* sp. monkeys, ECACC, No: 85020206) in Eagle’s minimum essential medium (EMEM, Life Technologies) in a 24-well plate. A 70 μl volume of each selected serum was first placed in a 56°C water bath for 30 minutes to inactivate the complement. To prepare the serial dilutions, 25 μl of each sample was mixed with 175 μl EMEM for a 1/8 dilution. Subsequently, 100 μl of this dilution was mixed with 100 μl EMEM for a serial two-fold dilution. The serial dilutions were mixed with 100 μl of virus suspension (40–80 plaque forming units) and incubated at 37°C for 1 hour before being added to the cells. The plates were incubated at 37°C for 1 hour before a carboxymethylcellulose (Sigma-Aldrich) overlay was added to each well.

After incubation, cells were fixed with buffered formalin, stained with Crystal Violet (Sigma-Aldrich) and the plates were placed on a light box to count the plaques and determine the PRNT. Samples were considered SBV positive if the 1/8 and 1/16 dilutions of the serum had plaque reductions of 95% (PRNT_95_) and 80% (PRNT_80_) respectively.

## Results

The SBV cELISA was carried out on 211 serum samples of Collection A, 28 of Collection B and 2 of Collection C from a total of 165 individual animals. The cELISA was positive for 68 samples from 48 individual animals representing 16 species from Collection A, 8 samples from 8 individual animals representing 7 species from Collection B, and for one of the 2 samples from Collection C.

Chronologically, prior to the first serologically positive case in Collection A, 103 samples of 95 individuals had tested negative. The first SBV positive at Collection A was a yak (*Bos grunniens*), sampled on August 21, 2012, at Collection B a roan antelope (*Hippotragus equinus*) sampled on September 27, 2012, and at Collection C an alpaca (*Vicuna pacos*), sampled on October 3, 2012. Since the first SBV positive in Collection A, 46 samples of 33 individuals tested positive and 24 samples of 23 individuals negative for SBV antibodies, giving SBV prevalence of 59% (Table 2). Three samples at Collection B screened negative prior to the first SBV positive case in September 2012. Since then, 8 samples of 8 individuals tested positive and 17 samples of 16 individuals negative, giving SBV prevalence of 33% for this collection.

**Table 2:** Samples on which a PRNT was carried out and/or those that had a positive cELISA result. The table includes animal species, date of sampling, cELISA results for each sample plus their corresponding S/N % values and PRNT results with antibody titres for a selection of the samples.

Date	Order—Species	cELISA (S/N%)—PRNT (Ab titre)
05/04/2011	Artiodactyla—*Bos grunniens*	NEG (55.7)—NEG
23/06/2011	Artiodactyla—*Oryx dammah*	NEG (52.2)—NEG
30/08/2011	Perissodactyla—*Ceratotherium simum*	Inc. (44.3)—NEG
24/01/2012	Artiodactyla—*Potamochoerus porcus*	NEG (80.1)—NEG
21/08/2012	Artiodactyla—*Bos grunniens*	POS (17)—NT
11/09/2012	Artiodactyla—*Bos grunniens*	POS (5.6)—NT
25/09/2012	Artiodactyla—*Camelus bactrianus*	NEG (51)—POS (1/16)
25/09/2012	Artiodactyla—*Tragelaphus spekii*	POS (27.7)—NT
25/09/2012	Artiodactyla—*Tragelaphus spekii*	POS (22)—NT
27/09/2012	Artiodactyla—*Tragelaphus spekii*	POS (6.4)—NT
27/09/2012	Artiodactyla—*Tragelaphus spekii*	POS (77.3)—NT
27/09/2012^a^	Artiodactyla—*Hippotragus equinus*	POS (20.5)—NT
02/10/2012^a^	Artiodactyla—*Tragelaphus eurycerus*	POS (10.8)—NT
03/10/2012^b^	Artiodactyla—*Vicugna pacos*	POS (10.8)—POS (1/256)
10/10/2012	Perissodactyla—*Equus hemionus*	POS (17.2)—NEG
16/10/2012	Artiodactyla—*Bos grunniens*	POS (5.5)—NT
24/10/2012	Artiodactyla—*Oryx dammah*	POS—8.2)—NT
26/10/2012	Artiodactyla—*Kobus megaceros*	POS (15.9)—NT
29/10/2012	Artiodactyla—*Tragelaphus strepsiceros*	POS (3.6)—POS (1/128)
30/10/2012^b^	Artiodactyla—*Lama glama*	NEG (65.1)—NEG
06/11/2012	Artiodactyla—*Alces alces*	POS (6.3)—NT
07/11/2012	Proboscida—*Elephas maximus*	POS (19.7)—NT
12/11/2012	Artiodactyla—*Capra aegagrus hircus*	POS (6.1)—NT
18/11/2012	Artiodactyla—*Bos grunniens*	POS (4)—NT
19/11/2012^a^	Artiodactyla—*Oryx dammah*	POS (3.6)—NT
29/11/2012	Artiodactyla—*Tragelaphus strepsiceros*	POS (4.5)—NT
03/12/2012	Artiodactyla—*Antilope cervicapra*	NEG (58.7)—NEG
08/12/2012	Artiodactyla—*Giraffa c*. *reticulata*	NEG (93)—NEG
31/12/2012	Artiodactyla—*Tragelaphus spekii*	POS (9.6)—NT
31/12/2012	Artiodactyla—*Tragelaphus spekii*	POS (7.7)—NT
16/01/2013	Artiodactyla—*Oryx gazelle gazella*	POS (4.8)—NT
04/02/2013	Artiodactyla—*Lama glama*	NEG (63.2)—POS (1/16)
06/02/2013	Proboscida—*Elephas maximus*	POS (15.5)—NT
13/02/2013^a^	Artiodactyla—*Syncerus caffer nanus*	POS (6.7)—NT
13/02/2013^a^	Artiodactyla—*Syncerus caffer nanus*	POS (6.2)—NT
07/03/2013	Perissodactyla—*Equus hemionus*	NEG (51.4)—NEG
16/03/2013	Artiodactyla—*Bos gaurus*	POS (15.8)—POS (1/32)
20/03/2013	Artiodactyla—*Oryx dammah*	POS (10)—NT
20/03/2013	Artiodactyla—*Tragelaphus spekii*	POS (13.8)—NT
21/03/2013	Artiodactyla—*Tragelaphus strepsiceros*	POS (5.2)—NT
09/04/2013	Artiodactyla—*Oryx dammah*	POS (3.6)—NT
21/04/2013	Proboscida—*Elephas maximus*	POS (13.7)—NT
24/04/2013	Artiodactyla—*Oryx dammah*	POS (4.7)—POS
30/04/2013	Artiodactyla—*Kobus megaceros*	POS (10.9)—NT
09/05/2013	Artiodactyla—*Tragelaphus spekii*	POS (6.5)—NT
15/05/2013	Artiodactyla—*Bos grunniens*	NT (NA)—POS (1/128)
15/05/2013	Artiodactyla—*Bos grunniens*	NT (NA)—POS (1/128)
15/05/2013	Artiodactyla—*Bos grunniens*	NT (NA)—POS (1/64)
19/05/2013	Proboscida—*Elephas maximus*	POS (18.1)—NT
21/05/2013	Artiodactyla—*Tragelaphus strepsiceros*	NT (NA)—POS (1/16)
23/05/2013	Artiodactyla—*Tragelaphus strepsiceros*	NT (NA)—POS (1/8)
25/05/2013	Proboscida—*Elephas maximus*	Inc. (42.7)—NEG
05/06/2013	Proboscida—*Elephas maximus*	POS (17.7)—NT
12/06/2013	Artiodactyla—*Rangifer tarandus*	POS (12.1)—POS (1/32)
12/06/2013	Proboscida—*Elephas maximus*	POS (21.1)—POS (1/32)
12/06/2013	Proboscida—*Elephas maximus*	POS (24.9)—NT
14/06/2013	Proboscida—*Elephas maximus*	POS (24.9)—NT
14/06/2013	Proboscida—*Elephas maximus*	POS (36.6)—NT
17/06/2013	Artiodactyla—*Bos grunniens*	POS (3.4)—POS (1/128)
17/06/2013	Artiodactyla—*Bos grunniens*	POS (3.3)—NT
17/06/2013	Artiodactyla—*Bos grunniens*	POS (3.3)—NT
18/06/2013	Proboscida—*Elephas maximus*	POS (15.7)—NT
18/06/2013	Proboscida—*Elephas maximus*	POS (18.5)—NT
20/06/2013	Artiodactyla—*Kobus megaceros*	NT (NA)—POS (1/64)
26/06/2013	Proboscida—*Elephas maximus*	POS (16.1)—NT
26/06/2013	Proboscida—*Elephas maximus*	POS (19.7)—NT
03/07/2013	Proboscida—*Elephas maximus*	POS (17)—NT
03/07/2013	Proboscida—*Elephas maximus*	POS (22.5)—POS (1/16)
07/07/2013	Artiodactyla—*Alces alces*	POS (4.3)—NT
10/07/2013	Proboscida—*Elephas maximus*	POS (20.1)—NT
10/07/2013	Proboscida—*Elephas maximus*	POS (17)—NT
10/07/2013	Proboscida—*Elephas maximus*	POS (20.8)—NT
10/07/2013	Proboscida—*Elephas maximus*	POS (20)—NT
11/07/2013	Artiodactyla—*Babyrousa celebensis* ^a^	POS (3.7)—POS (1/64)
26/07/2013	Proboscida—*Elephas maximus*	POS (29.3)—NT
30/07/2013	Artiodactyla—*Hyelaphus porcinus*	NEG (75.2)—NEG
13/08/2013	Artiodactyla—*Babyrousa celebensis* ^a^	NEG (81.7)—NEG
14/08/2013	Artiodactyla—*Bos grunniens*	POS (3.2)—NT
14/08/2013	Artiodactyla—*Bos grunniens*	POS (3.8)—NT
28/08/2013	Perissodactyla—*Equus hemionus*	NEG (52.7)—NEG
03/09/2013	Artiodactyla—*Rusa alfredi* ^a^	POS (3.9)—NT
09/09/2013	Artiodactyla—*Bos grunniens*	POS (7.7)—NT
11/09/2013	Artiodactyla—*Bison bonasus*	POS (15.5)—NT
19/09/2013	Artiodactyla—*Bison bonasus*	POS (7.8)—NT
21/09/2013	Perissodactyla—*Equus grevyi*	POS (37.6)—NEG
27/09/2013	Artiodactyla—*Bison bonasus*	POS (6.8)—NT
02/10/2013	Artiodactyla—*Oryx dammah*	POS (9.7)—NT
02/10/2013	Artiodactyla—*Oryx dammah*	POS (4.9)—NT
02/10/2013	Artiodactyla—*Oryx dammah*	POS (3.3)—NT
02/10/2013	Artiodactyla—*Oryx dammah*	POS (15.5)—NT
02/10/2013	Artiodactyla—*Oryx dammah*	POS (4.2)—NT
02/10/2013	Artiodactyla—*Oryx dammah*	POS (5.4)—NT
02/10/2013	Artiodactyla—*Oryx dammah*	POS (4)—NT
30/10/2013	Artiodactyla—*Hyelaphus porcinus*	NT (NA)—NEG
22/11/2013	Artiodactyla—*Tragelaphus eurycerus*	POS (22.3)—NT
27/11/2013	Artiodactyla—*Bos javanicus* ^a^	POS (30.9)—NT
11/12/2013	Artiodactyla—*Dama dama*	NT (NA)—POS (≥48)
15/01/2014	Perissodactyla—*Equus hemionus*	NEG (56.5)—NEG
12/02/2014	Artiodactyla—*Giraffa c*. *reticulata*	POS (23.1)—POS (1/16)

NT; not tested.

NA; not available.

Inc.; inconclusive results.

^a^Collection B.

^b^Collection C.

All Asian elephants (*Elephas maximus*) at Collection A sampled (n = 8) in August 2012 were negative on the cELISA. Follow-up serial serum samples were available for six of these elephants. A juvenile male elephant sero-converted between September and November 2012. The breeding bull sero-converted between September 2012 and June 2013. One female elephant tested inconclusive in May, but positive in July 2013 on the cELISA. One female and her juvenile female offspring sero-converted between August 2012 and June 2013. One female did not sero-convert. Sera from two Asian elephants at Collection B obtained respectively in July and September 2013 were negative.

SBV sero-positives also included recent imports into Collection A; three yaks from the Netherlands (first sampled a week after arrival on May 15, 2013 and on June 17, 2013) and a greater kudu (*Tragelaphus strepsiceros*) from France (sampled on May 23, 2013, before moving to Whipsnade zoo in June 2013).

Serial sampling at Collection A confirmed sero-conversion in 21 individuals from 11 different species: one greater kudu, two scimitar-horned oryx (*Oryx dammah*), three yaks, one gemsbok (*Oryx gazelle gazella*), one reticulated giraffe (*Giraffa camelopardalis reticulata*), one llama (*Lama glama*), one moose (*Alces alces*), two European bison (*Bison bonasus*) and three female sitatunga (*Tragelaphus spekii*) (Table 3). Serial blood samples of a female pygmy hippopotamus (*Hexaprotodon liberiensis*) over a similar period did not reveal SBV sero-conversion.

**Table 3:** First SBV seroconversion detected testing serial blood samples from 14 individuals of 10 animal species from Collection A using cELISA.

Species	Sex	Last negative	First positive	Subsequent positive
Asian elephant	male^a^	Sep. 2012	Nov. 2012	July 2013
Asian elephant	male	Sep. 2012	June 2013	NA
Asian elephant	female	May 2013^b^	July 2013	NA
Asian elephant	female	Aug. 2012	June 2013	NA
Asian elephant	female^a^	Aug. 2012	June 2013	July 2013
Greater kudu	male	Nov. 2011	Nov. 2012	May 2013
Scimitar-horned oryx	female	Jan. 2011	Oct. 2012	Oct. 2013
Scimitar-horned oryx	male	July 2011	April 2013^c^	NA
Yak	male	June 2011	Sep. 2012	NA
Yak	female	Jan. 2012	Nov. 2012	NA
Yak	female	Feb. 2012	Nov. 2012	NA
Gemsbok	male	June 2011	Jan. 2013	NA
Reticulated giraffe	male	May 2012	Feb. 2014	NA
Llama	male	July 2011	Feb. 2013	NA
Moose	male	Aug. 2011	July 2013	NA
European bison	female	June 2011	Sep. 2013	NA
European bison	female	June 2011	Sep. 2013	NA
Sitatunga	female	Dec. 2011	Sep. 2012	NA
Sitatunga	female	April 2012	Mar. 2013	NA
Sitatunga	female	Mar. 2011	May 2013	NA

NA; not available.

^a^Juvenile elephants.

^b^Inconclusive.

^c^The cELISA result was also confirmed by PRNT.

PRNT was carried out on 35 samples from 17 individuals representing 18 different species from Collection A and on 2 samples from one species from Collection B. There was complete concordance between the PRNT and cELISA results for deer (hog deer *Axis porcinus*, reindeer *Rangifer tardinus*), antelope (greater kudu, blackbuck *Antelope cervicapra*), bovids (yak, gaur *Bos gaurus*), giraffes and red river hogs (*Potamochoerus porcus*). There was also concordance between the positive and negative outcomes of the cELISA and PRNT for Asian elephants. One inconclusive cELISA result from an Asian elephant (S/N% = 42.7) was negative on PRNT.

The cELISA and PRNT results did not correlate for the serum samples tested from camelids and perissodactyls. Of the three positive PRNT results in camelids (an alpaca, a llama and a Bactrian camel *Camelus bactrianus*), only the alpaca, with the highest PRNT titre of these three camelids, produced a positive cELISA result. Forty-two perissodactyl serum samples from 27 individuals of 9 species were screened. One onager (*Equus hemionus*) and a Grevy’s zebra (*Equus grevyi*) were positive on cELISA but not on PRNT, although two out of four individual onagers screened showed some plaque reduction at the initial serum dilution. One further cELISA inconclusive result (a male white rhinoceros, *Ceratotherium simum*) was also PRNT negative with an insufficient plaque reduction (<90%) at the 1/8 dilution.

## Discussion

To our knowledge this is the first systematic evaluation of previous SBV infection in exotic ungulates in Europe. The study provides compelling evidence of SBV infection in exotic *Bovidae*, *Cervidae*, *Suidae*, *Giraffidae* and most notably in Asian elephants (*Elephas maximus*)–one of the endangered flagship ungulate species [18]. Serial serum samples of several individuals suggest that ungulates at Collections A, B and C were first exposed to SBV in the late summer of 2012, the second year of SBV circulation in the UK. Whilst this is generally in line with the spread of this virus to livestock holdings across the UK [19], both ZSL collections were in geographical areas where the first incursion of SBV occurred in 2011. Notably, some animals, including one of the Asian elephants, sero-converted only in 2013, the third SBV season in the UK, demonstrating the continuous spread of SBV in an affected area of the UK (South East) for three consecutive years. Interestingly, in this context and in relation to the biology of the *Culicoides* vector midges, all the positive cases at Collection A were animals housed near buildings or which preferred to shelter under bushes and/or trees. Most species in the ‘Drive Through’, an exposed wide open space with limited housing and vegetation, were negative for SBV. Examples are Chinese water deer (*Hydropotis inermis*), hog deer and Pere David’s deer (*Elaphurus davidianus*). Other studies have also shown that landscape elements and shelter can influence the spatial distribution of *Culicoides*-borne diseases [20–21], likely through a variation of exposure time to the midges.

A previous study comparing an indirect ELISA (iELISA) with serum neutralisation test (SNT) on a large number of red deer sera confirmed the reliability of using iELISA for this species [22]. Similar conclusion could be drawn on the use of the cELISA for the majority of species screened in this study, with the exclusion of camelids and perissodactyls. However, it has to be taken into consideration that only a small number of individuals of each species were tested in this study.

SBV exposure of camelids has previously been established using an iELISA [23]. However, in this study the cELISA proved unsuitable for the screening of camelids, resulting in false negatives in both a llama and a Bactrian camel. This was unexpected as SBV cELISA performance in other ruminants, including wild deer and bison, is considered to be optimal [22]. However, this study only examined a handful of camelid samples and hence cELISA appliance in camelids requires further validation. In the meantime, virus neutralisation assays and iELISA should be considered as the preferred tests for the screening of camelids.

As far as perissodactyls are concerned, a fair number of sera returned inconclusive (white rhinoceros) or positive (onager, Grevy’s zebra) results on the cELISA, which could not be verified by the PRNT. This variability and, at times, marked plaque reduction in the initial dilution potentially points towards an SBV-related virus, against which the antibodies cross-react in the cELISA. Similar observation has also been made testing horse sera for SBV antibodies (unpublished data). Further research is therefore required to resolve these inconsistencies.

Following the first serologically positive yak at Collection A in August 2012, almost two thirds (59%) of individual artiodactyls at Collection A tested positive for SBV antibodies. This is similar to in-herd sero-prevalence findings in livestock following initial infection [24]. Screening of the large number of serial serum samples from the Asian elephant herd at Collection A showed that the majority of animals within an exposed herd sero-converted within a similar time frame, and that, after initial exposure, antibody levels remained high. Similar trends were also seen in serial serum samples from other species, including a greater kudu and a scimitar-horned oryx, in which the antibody level remained high for at least 6 to 12 months, respectively. This is consistent with persistent antibody levels seen in cattle [24].

As yet, clinical evidence of the presence of SBV has not been seen at Collections A, B or C. Transient fever, impaired general condition and diarrhoea, as seen in cattle [25], may have been missed in exotic ungulates due to the nature of their hands-off management. However, as yet an increase in congenital deformities and/or abortions has not been observed at any of the three sites studied. Routine tissue testing at Collection A of several aborted foetuses by PCR has also yielded negative results (unpublished data). As SBV antibodies are considered protective [26–27] disease is only expected in naïve animals or those that have not sero-converted [28]. Therefore both clinical and economical impacts at zoos can be considered negligible even for naïve populations. In line with the notion that previous SBV infection (documented through the presence of antibodies) protects from re-infection for a considerable length of time, no clinical signs consistent with SBV infection have been observed in any of the animals studied at these zoological collections.

Further research is currently taking place on the vector presence, infection rate and seasonality at Collection A, in an attempt to gain insights into potential correlation between seasonality of virus load in vectors and sero-prevalence. Based on such further findings, conclusions may potentially be drawn as to the value of vaccination of exotic ungulates against SBV.
